# Intelligent metasurface with frequency recognition for adaptive manipulation of electromagnetic wave

**DOI:** 10.1515/nanoph-2021-0799

**Published:** 2022-03-07

**Authors:** Hai Peng Wang, Yun Bo Li, He Li, Jia Lin Shen, Shu Yue Dong, Shi Yu Wang, Kai Nan Qi, Qian Ma, Shi Jin, Si Jia Li, Tie Jun Cui

**Affiliations:** State Key Laboratory of Millimeter Waves, Southeast University, Nanjing, 210096, China; Science and Technology on Electromagnetic Scattering Laboratory, Beijing, 100854, China; National Mobile Communications Research Laboratory, Southeast University, Nanjing 210096, China; Information and Navigation College, Air Force Engineering University, Xi’an, 710077, China

**Keywords:** adaptive intelligent metasurface, control of spatial electromagnetic wave, customized multifunction, frequency recognition

## Abstract

Due to the strong ability of recognizing electromagnetic (EM) environment and adaptively control of EM waves, the intelligent metasurfaces have received great attention recently. However, the intelligent metasurface with frequency recognition for adaptive manipulation of the EM waves has not been studied. Here, we propose a frequency-recognition intelligent metasurface to precisely control the spatial EM waves under the agile frequencies with the help of a real-time radio-frequency sensor and an adaptive feedback control system. An active meta-atom is presented to reach 2 bit phase coding and 1 bit amplitude coding capacities to control the amplitude and phase independently. Experimental results demonstrate that the metasurface can recognize different frequency of the incoming wave with very high resolution, and can adaptively realize the self-defined multiple frequency agilities to manipulate the reflected EM waves without any human participation. As example, the intelligent metasurface with frequency recognition can adaptively operate wave absorption at 5.36 GHz, reflection to normal direction at 5.38 GHz, deflection to −30° at 5.40 GHz, random diffusion at 5.42 GHz, and deflection to +33° at 5.44 GHz by detecting the incoming frequency at the resolution of 0.02 GHz.

## 1 Introduction

Metasurfaces have attracted a great deal of attention owing to their advantages of low loss, easy fabrication, low profile and multi-versatility [1], [2], [3], [4], [5], [6], [7], [8]. They are defined as the two-dimensional (2D) structures at surfaces or interfaces with different geometries and distributed functional arrangements. Since the generalized Snell’s laws of reflection and refraction [9] was introduced to provide full control of the wavefront by spatially tailoring the metasurfaces with abrupt phase changes, the gradient-phase metasurfaces have opened up an unprecedented venue for artificially manipulating the EM waves, such as vortex beam shaping [10, 11], invisibility cloaking [12], [13], [14], broadband achromatic metalensing [15], optical holography [5, 16], foucsing [17, 18] and the converting from spatial wave to surface wave [19]. Recently, owing to the simultaneous manipulations of EM waves in both the space and frequency domains, the space-time-coding digital metasurfaces [20] and time-domain digital coding metasurfaces [21, 22] have attracted growing interests and provided promising applications in multi-user new-architecture wireless communications, cognitive radar, and related areas.

With the rapid development of various tuning strategies, a great deal of tunable or reconfigurable metasurfaces have been proposed to achieve in dynamic manipulation of EM waves to solve the drawback of static functionality that depending on their fixed geometrical parameters in the passive metasurfaces [23]. In the microwave band, by integrating discrete active elements (e.g. varactors [24], [25], [26], [27], [28], [29], [30], [31], PIN diodes [32], [33], [34], [35], [36] and amplifiers [37]) in the meta-atom, metasurfaces can realize reconfigurable EM response. The absorption frequency can be shifted through feeding the reverse bias voltage on the loaded varactors in the tunable metasurface [24, 25], and the recent work using metasurfaces on perfect absorption can achieve reflection values around −80 dB [25]. The electrically reflection [26] and transmission [27] tunable metasurfaces have been presented to realize the polarization converter and rotator. The beam-forming performance (including wide bandwidth, wide scanning range and low insertion loss) can be improved using various antennas based on reflectarrays [32] and transmitarrays [28, 29], where the formed unit cells providing a continuous electrically reconfigurable phase range of 360°. A spatial-energy digital-coding metasurface [37] with active amplifiers was proposed to realize arbitrary editing of the energy of spatial propagating waves. However, most of the tunable metasurfaces can only achieve similar response of some function in a special frequency band, e.g., absorption frequency shifting, polarization states rotating, and beam-scanning angle adjusting.

In the microwave regime, more attentions have been focused on the design of multifunctional metasurface [38, 39]. Two different wave-manipulation functionalities of focusing and beam bending have been realized by using passive anisotropic metasurfaces [38] with polarization-dependent phase responses. Since the digital coding and programmable metasurfaces [33] have been introduced to dynamically manipulate different scattered EM wave modulations (including beam deflection, multi-beam and reduction of radar cross-sections), some programmable and reconfigurable metasurfaces [30, 34], [35], [36] have been enormously promoted due to the multifunctional control of the EM waves. Huang et al. have designed a reconfigurable transmitarray [30] and a reflectarry [34] with capabilities of dynamical beam steering and polarization transformation. In addition, the switchable transmission-reflection response [36] with different polarizations was reported by using a reconfigurable anisotropic digital coding metasurface. Some work related to reconfigurable intelligent surfaces. In the area of intelligence and biosensing, the programmable metasurfaces with electronically controlled PIN diodes have been employed to demonstrate a real-time intelligent microwave imager, body gestures recognizer and vital-sign monitor, empowered by machine-learning optimized methods and artificial neural networks [40], [41], [42].

Recently, the development of adaptive variants, in which the EM properties dynamically vary in response to external environment or stimulus [43], has emerged as a significant scientific research hotspot. Therefore, some smart sensing metasurfaces with self-adaptively and self-tuning capability [44], [45], [46], [47], [48] are introduced. The adaptive metasurfaces contain sensing and feedback parts to constitute a closed-loop system, so that the active devices can be self-tuned to control the EM wavefronts without any manual control or intervention. Cui et al. put forward a metasurface integrated with a gyrocope sensor and a feedback system [44], which can self-adaptively adjust the coding pattern to steer the radiation beam towards the fixed spatial location under different rotations of metasurface without human paticipaiton. In addition, an intelligent microwave invisibility cloak [45] was developed by using two detectors (incident angle and reflected spectrum) and a tunable metasurface with the aid of deep-learning-driven method. The metasurface cloak can respond to incident wave and surrounding environment in real-time without human intervention. Furthermore, other adaptive metasurfaces manipulating the microwave responses, have also been proposed in the sensing form of radio-frequency (RF) power [46] and thermal [47, 48], depending on the application scenario.

However, to the best of the knowledge, the frequency diversity has valuable applications, but the intelligent EM metasurface with frequency recognition for adaptive manipulation of spatial EM waves has not been extensively studied. Due to the advantage of frequency dispersion, a microstrip-based meta-imager was presented to be capable of imaging under compressed sensing at microwave frequencies [49]. The foundations are the apertures based on random holographic leaky-wave metasurface. It can be considered as a single-sensor imaging system via a series of frequency-diverse and orthogonal measurements with a single frequency sweep, obviating mechanical scanning and multiple detectors [50]. Then two practical holographic imaging systems were demonstrated to accelerate the microwave scene process and to provide complex-object imaging [51, 52]. However, the conventional leaky-wave antennas [53, 54] can be used to generate a directed beam whose radiation angle monotonously varies with the input frequency, and only have fixed function of frequency-scanning.

In this paper, we introduce and demonstrate experimentally a frequency-recognition intelligent metasurface system operating in the microwave frequencies for self-adaptive precise control of spatial EM reflection spectrum under agile frequency. To implement this smart system, an active reflective metasurface with independent control of amplitude and phase is carefully designed with a radio frequency sensing module and an adaptive feedback control module. The created intelligent frequency-recognition metasurface system is able to realize the self-adaptive multiple frequency agilities for the space controls of reflected EM waves, including absorption, reflection and scattering pattern control at fixed frequency without any human intervention. According to the simulations and experimental measurements, the results demonstrate a great capability of adaptive and accurate reflective wavefront manipulation with recognizing frequency in quite high frequency resolution. This verifies that the frequency-recognition metasurface can provide perfect self-adaptability with self-designed functions in response to different frequencies of incident waves.

## 2 Results and discussion

### 2.1 Architecture of the intelligent metasurface system

**Figure 1:**
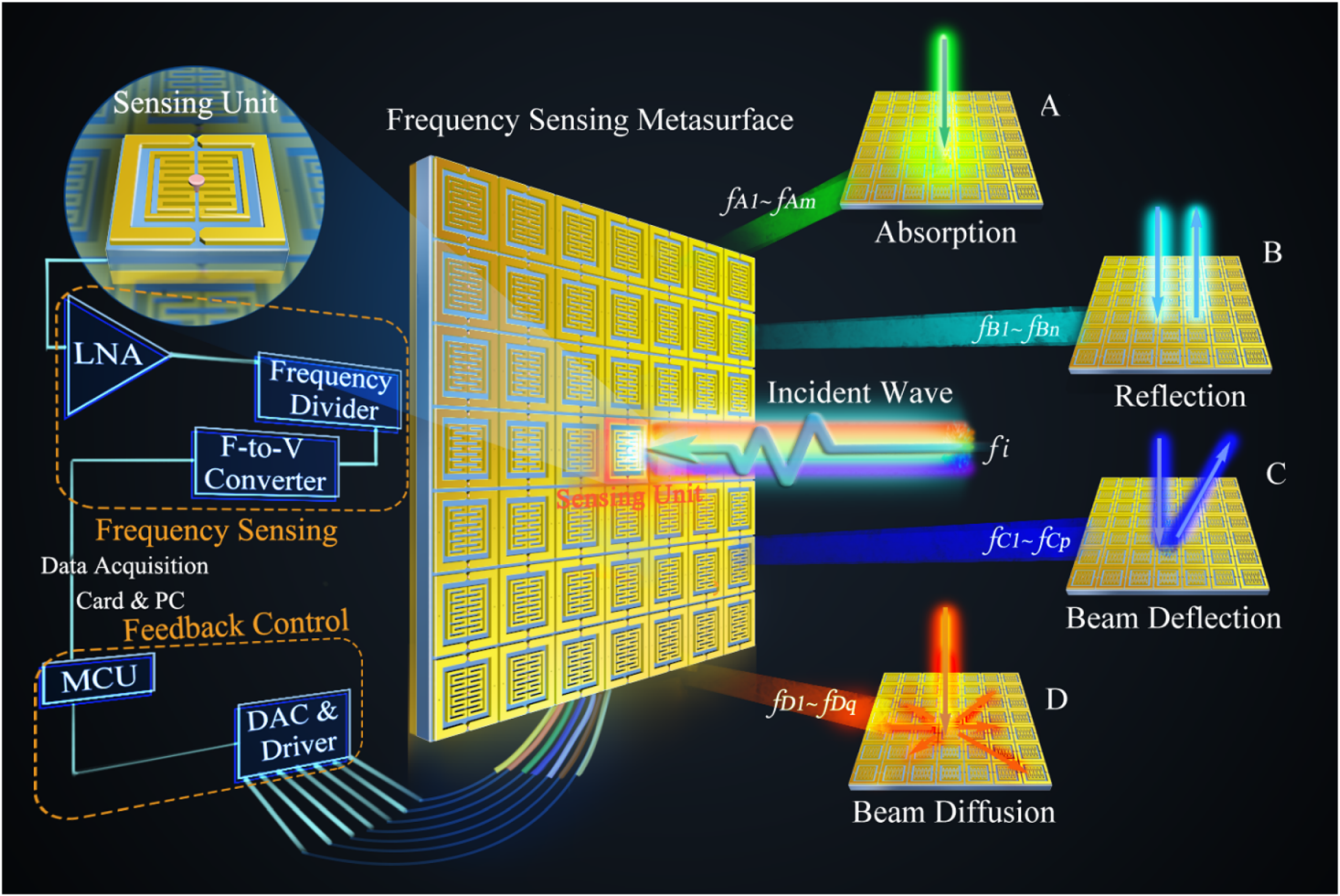
Schematic of the frequency-adaptive intelligent metasurface for self-adaptively precise control of spatial EM reflection spectrum. It consists of a programmable reflective metasurface with independent control of amplitude and phase, a real-time radio frequency sensing and adaptive feedback control electronic system. The intelligent metasurface system is able to adaptively implement the customized functions to match the corresponding frequency of the incoming waves. For example, absorption can be achieved when detecting the frequency of incident wave at *fA*_1_, …, *fA*_*m*_, while the reflection, beam deflection and diffusion can be realized when sensing the frequency at *fB*_1_, …, *fB*_*n*_, *fC*_1_, …, *fC*_*p*_, and *fD*_1_, …, *fD*_*q*_, respectively. The above frequencies mentioned are detected with extremely high frequency resolutions.

Generally, the artificial EM functions designed by metasurfaces all have a certain working bandwidth. Therefore, in the case of unattended condition, which means under the premise of no active control and human intervention, it is impossible to realize diverse violent alternated performance for the space controls of electromagnetic waves in a highly narrow frequency band. The mentioned property is very interesting and has greatly practical application value. For example, it can be implemented in the EM stealth, camouflage and electronic warfare through using the violent agilities in the frequency spectrum. Also, this feature can be adopted to design multifunctional spectrum multiplexing in the communication systems. To realize this goal, the metasurface should be designed with the capability of recognizable and adaptive, which means that it can sense the frequency of incoming waves and then automatically feedback and adjust to meet the functional design requirements. As shown in Figure 1, we present a frequency-recognition intelligent metasurface for self-adaptively control of spatial EM waves. The only one sensing element in the central position of the proposed metasurface, integrating with radio-frequency sensing module, is responsible for receiving and measuring the frequencies of incident EM waves. Under the operation of the adaptive feedback control module, all the bias voltages are automatically calculated and immediately provided to the metasurface. Therefore, after recognizing different frequencies of incoming waves, the intelligent metasurface system is able to adaptively implement the customized functions such as absorption, reflection, beam deflection and diffusion. In order to implement the corresponding function of feedback adjustment, the reflection amplitude and phase response of each active meta-atom should be independently tuned by regulating the direct-current (DC) bias voltage across the embedded varactors in the metasurface at microwave frequencies.

### 2.2 Reconfigurable reflective meta-atom with independent control of phase and amplitude

**Figure 2:**
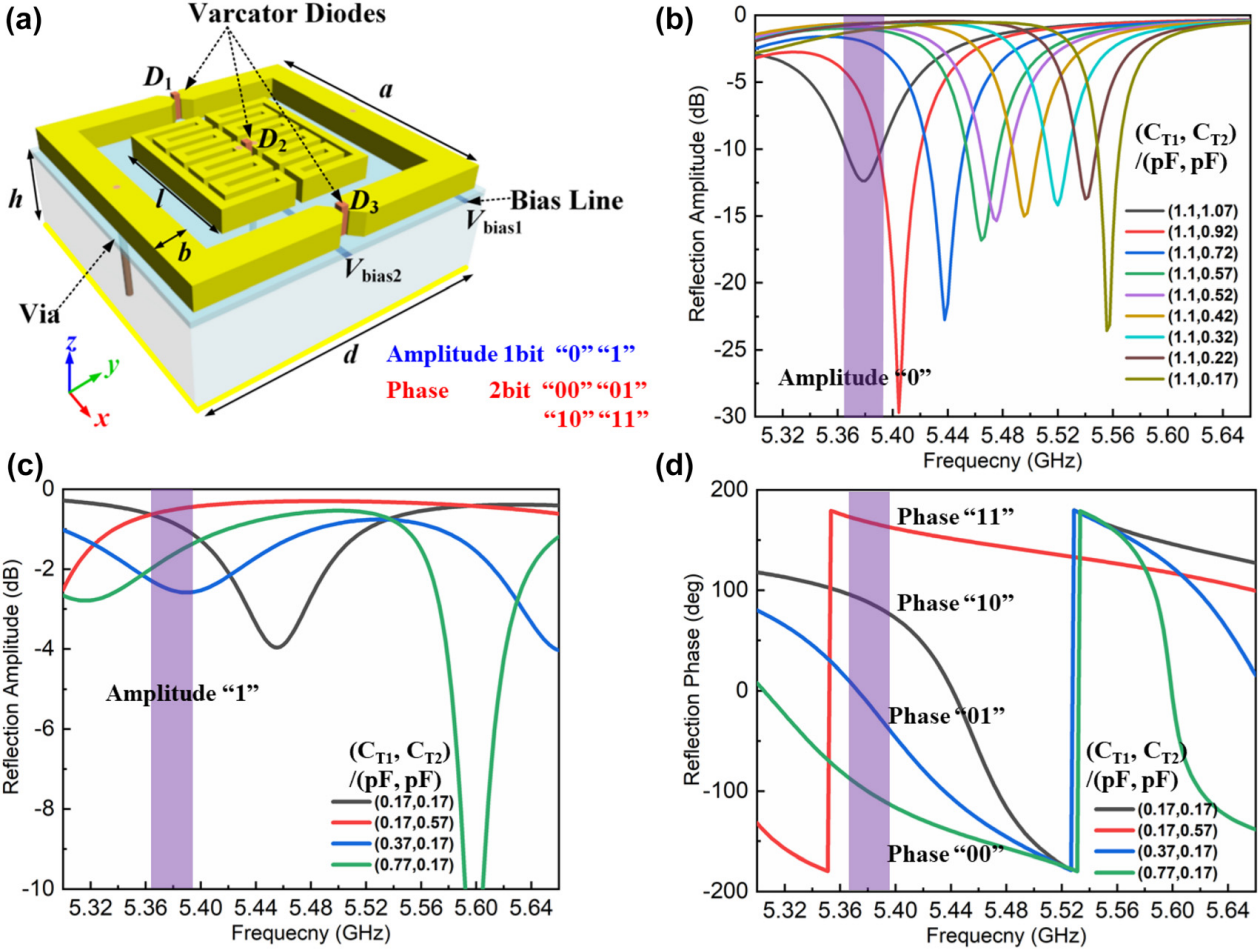
Designed reconfigurable meta-atom with independent reflective amplitude and phase response. (a) Three-dimension illustration of the meta-atom (unit cell) with three identical varactors. (b) Simulated reflection amplitude ‘0’ curves of the meta-atom for different capacitances (C_T1_, C_T2_) under *y*-polarized incidence at the frequency range of 5.36–5.56 GHz. (c) and (d) Simulated reflection amplitude ‘1’ curves with 2 bit phase response curves for different capacitances (C_T1_, C_T2_) under *y*-polarized incidence at the frequency of 5.38 GHz.

In order to simultaneously implement the customized functions such as absorption, reflection, beam deflection and diffusion, it is necessary to design a reflective metasurface with independent control of amplitude and phase. We propose an active meta-atom with a specially designed top metal pattern and three varactors, as illustrated in Figure 2(a). The top metal pattern of the unit cell is composed of a three-ring structure, in which the outer ring is a split ring and two inner rings are closed rings. Two identical metallic split-square-ring are symmetrically placed on top of the dielectric substrate to construct the outer ring, with two microwave varactor diodes (D_1_, D_3_) paralleled connected between the shorter arms. Similarly, two identical inner rings are symmetrically laid on the on top of the substrate with loading a varactor (D_2_). For the resonant reflection unit, the reflection phase can be mainly tuned by changing the capacitance of the varactors loaded between the outer ring, and the diode loaded at the inner rings is responsible for adjusting the reflection amplitude. We remark that the metallic square rings with meander line are selected as the inner rings, so that it can effectively increase the equivalent inductance to be consistent with the outer ring. Therefore, it can keep the phase and amplitude control in the same frequency band. Details on the unit cell and simulations are provided in Supplementary note 1.

The EM performance of the designed meta-atom is examined numerically. C_T1_ represents the capacitance value of varactor D_1_ and D_3_, and C_T2_ represents the capacitance value of varactor D_2_. The reflection spectrum can be simulated under the sweep of capacitances configuration (C_T1_, C_T2_). The simulated reflection amplitude ‘0’ curves of the meta-atom for different capacitances under the y-polarized incidence are shown in Figure 2(b). It is obvious that the resonant frequency shifts from 5.36 GHz to 5.56 GHz as the capacitance C_T2_ adjusts from 0.17 pF to 1.1 pF, while the capacitance C_T1_ keeps nearly constant value. It can be verified that varactor D_2_ loaded at the inner rings has main contribution for adjusting the resonant frequency. The reflection amplitude ‘1’ curves with 90° phase difference response at 5.38 GHz are simulated with four different capacitances configurations (Figure 2(c) and (d)). The four capacitance configurations are corresponding to the four phase coding states ‘00’, ‘01’, ‘10’, and ‘11’, respectively. The reflection amplitudes are larger than –3 dB and the range of amplitude variation is below −2 dB in the frequency range between 5.37 and 5.39 GHz. Although it is a narrow band, we can realize the 2 bit phase response with amplitude ‘1’ at some frequency through adjusting appropriate voltages configurations of varactor diodes.

### 2.3 Adaptive metasurface with frequency recognition

**Figure 3:**
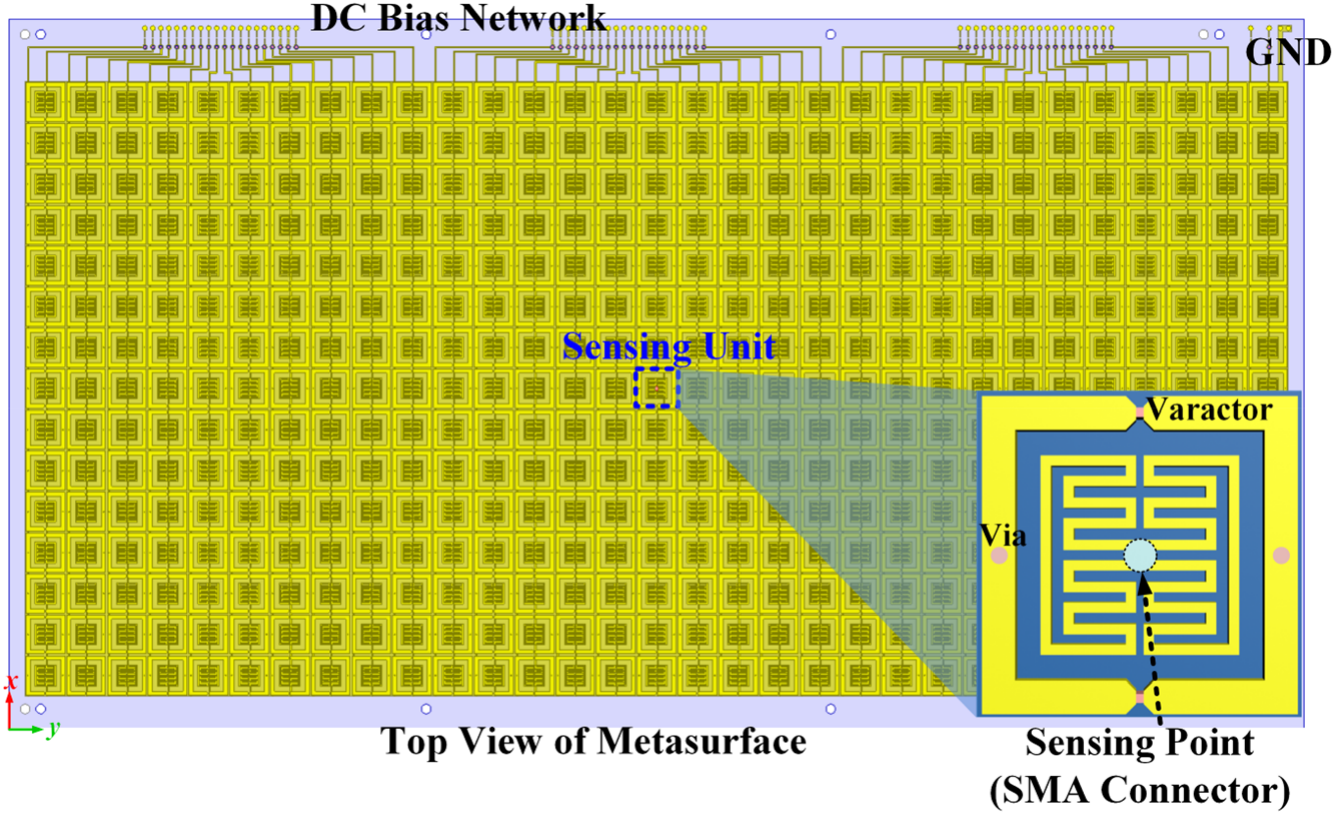
Top view of the intelligent metasurface with frequency sensing and the bias network. The central unit (inset) is sensing the incident wave and is connected with frequency sensing module.

The designed metasurface is composed of 31 × 15 meta-atoms (∼7.33*λ* × 3.55*λ*) (Figure 3). Each column consists of 15 meta-atoms and shares the same bias voltage configuration in the 2D cases, except the central unit for recognizing the incident wave. As shown in the inset of Figure 3, in order to be consistent with the surrounding meta-atoms and can simultaneously absorb the energy of incident wave, a sensing point (a via hole with diameter 1.3 mm) is designed to replace the varactor and solder with a subminiature version A (SMA) connector, which is in the gap between two inner rings with meander line structure. Thus, the energy of the spatial incidence captured by the two metallic inner patches in the sensing unit goes through the frequency sensing module via the SMA connector.

**Figure 4:**
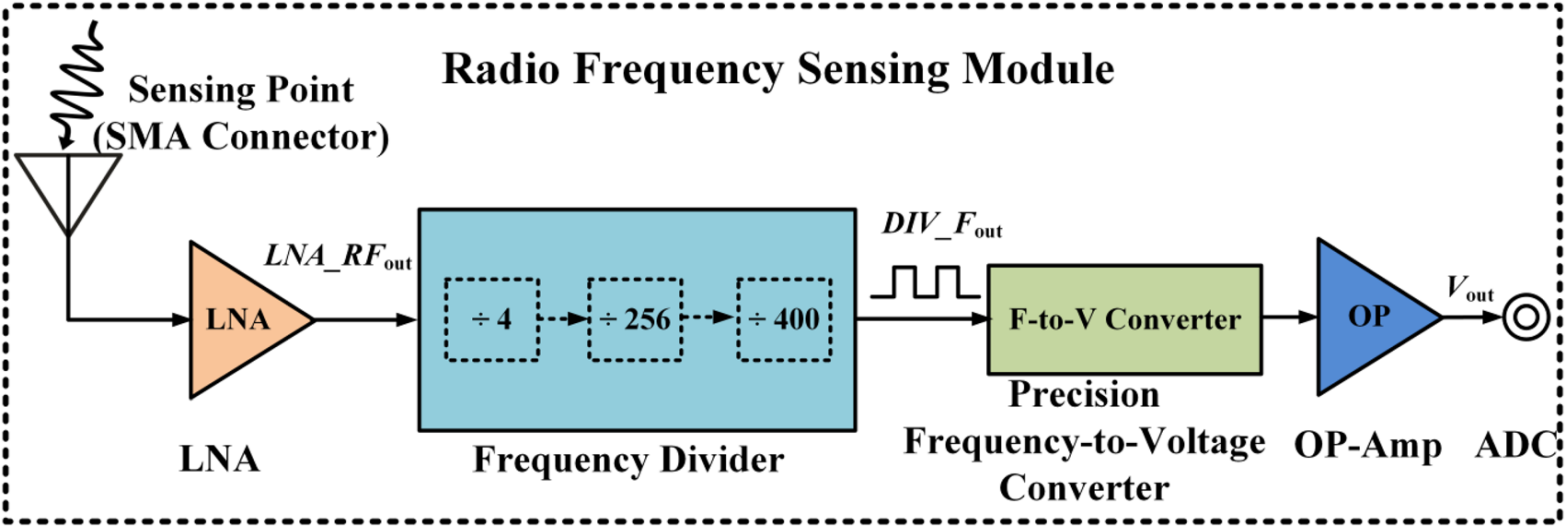
Block diagram of the radio frequency sensing module. It is mainly composed of a LNA, three frequency dividers, a precision frequency-to-voltage converter and an ADC.

**Figure 5:**
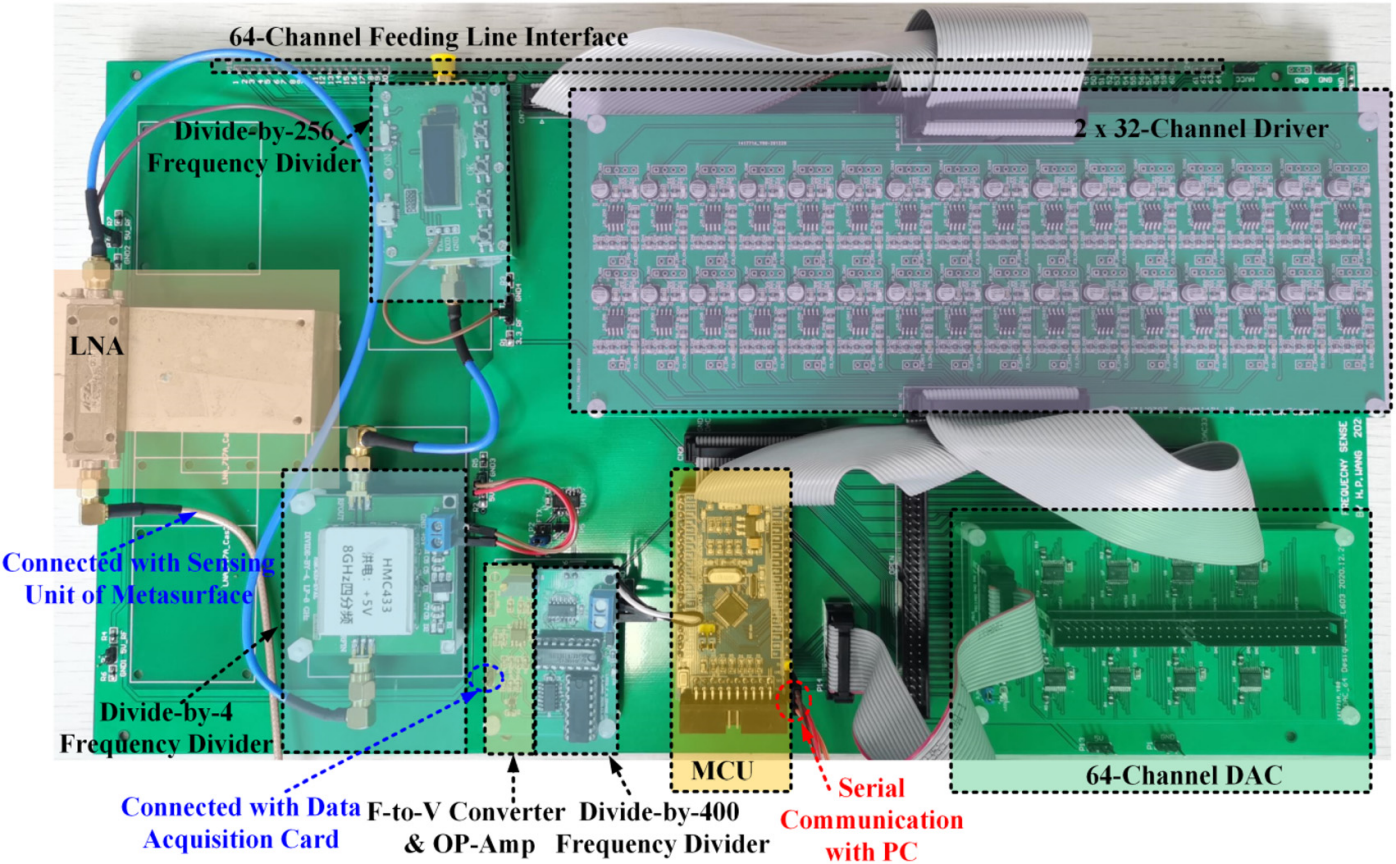
Photo of designed frequency sensing and adaptive feedback control electronic system. The frequency sensing and adaptive feedback control electronic system and the metasurface sample are fixed together through plastic screws.

The connected frequecy sensing module is used for detecting the frequency of incident EM waves (Figure 4). It is mainly composed of a low noise amplifier (LNA), three frequency dividers, a precision frequency-to-voltage converter and an analog-to-digital converter (ADC). The final output of the frequency sensing module is a DC voltage signal and has a linear relationship with the frequency of the incident wave. Hence, the frequency information of incoming waves can be obtained by using the ADC. Here we use a data acquisition card and the LabView software running in the personal computer (PC), and the frequency information is transmitted to a micro-controller unit (MCU) through the serial communication. The MCU is the core of the adaptive feedback control module, and it is used to distribute all commands to the varcator diodes through the programmable DC sources according to the frequency information. The programmable DC sources are composed of digital-to-analog converters (DACs) and the corresponding driver circuits. Thus, the metasurface can be dynamically controlled and switched by simply changing the varactors configuration through the programmable DC sources in the adaptive feedback control module. Therefore, the proposed metasurface system can adaptively alter the customized functions to match the recognized frequency of the incoming waves. In addition, we remark that our designed frequency sensing module can detect the frequency band from 5 to 6 GHz with a quite high frequency resolution accuracy of 0.02 GHz, and the customized EM functions under the agile frequencies can be achieved in quite high resolution with adjusting appropriate voltage configurations to realize independent control of 2 bit reflection phase coding and 1 bit amplitude coding. Figure 5 shows the photo of designed frequency sensing and adaptive feedback control electronic system. Details on the circuit design, test of frequency sensing module and adaptive feedback control module are provided in Supplementary notes 2 and 3.

### 2.4 Experiment results and discussion

#### 2.4.1 Results of meta-atom using standard waveguide

Before the metasurface sample is fabricated, the meta-atom should be experimentally tested through waveguide to verify the reflection characteristics. Details on the experimental setup and results of the meta-atom using standard waveguide is provided in Supplementary note 4. The test results of waveguide show that the designed meta-atom can realize the 2 bit phase and 1 bit amplitude response at a variable frequency through adjusting the DC bias configurations. Therefore, the DC bias voltages loaded at the varactors are required to be calibrated before the metasurface sample is implemented for the self-designed EM functions at corresponding frequency of the incident wave.

#### 2.4.2 Metasurface system fabrication

**Figure 6:**
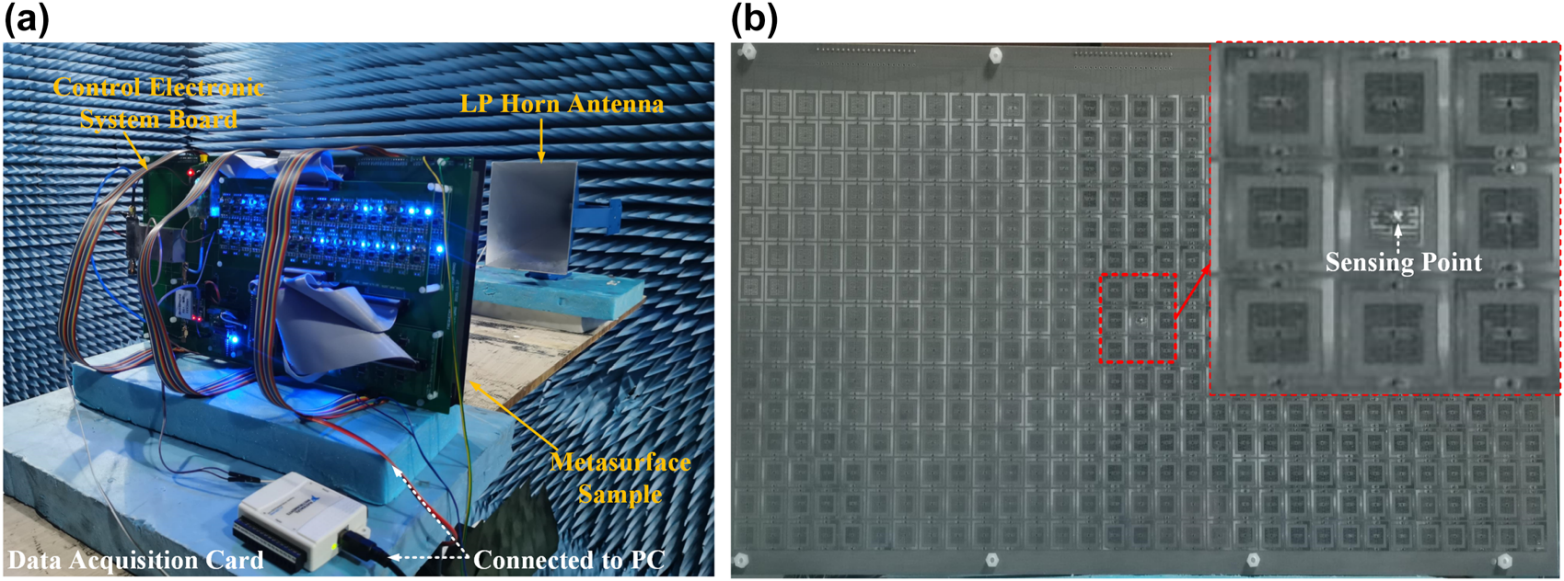
Experimental setup. (a) and (b) Photographs of the experimental setup in an anechoic chamber and the fabricated prototype, respectively. The sensing unit and its surrounding eight meta-atoms are enlarged in the inset.

Based on the above design, we fabricate a prototype of the proposed metasurface with the frequency sensing and adaptive feedback control electronic system, as shown in Figure 6(b). The metasurface sample composed of 31 × 15 coding elements is fabricated using printed circuit board technology. Each column of the metasurface is composed of fifteen coding elements and shares a common control voltage. The size of the coding element is 13 × 13 mm^2^, corresponding to about 0.24*λ*_c_ × 0.24*λ*_c_ at the center operational frequency of 5.46 GHz. The overall size of the fabricated metasurface sample is 413 × 225 mm^2^ (7.52*λ*_c_ × 4.10*λ*_c_). Then the 1394 varcator diodes and an SMA connector were machined-weldinged into the metasurface sample. Finally, the metasurface sample with the frequency sensing and adaptive feedback control electronic system were fixed together through plastic screws.

#### 2.4.3 Experimental setup and calibration of DC voltages for the metasurface sample

The experiments are carried out in a microwave anechoic chamber, as illustrated in Figure 6(a). In the measurement, two linearly polarized (LP) horn antennas working from 4.6 to 7 GHz were employed as the transmitting (TX) and receiving (RX) antennas, respectively. The fabricated prototype is illuminated by the TX horn antenna placed along the normal incidence, and the distance between the prototype and TX horn was 70 cm. Both the metasurface system and the TX horn antenna are mounted on a turntable. The DC bias voltages loaded at the varactors are required to be calibrated before the experiment. The programmable voltage source instrument (NI PXIe-6739R) is used to provide dynamic biasing voltages for the calibration of coding metasurface. The 62 bias channels are (*V*_bias1_, *V*_bias2_, *V*_bias1_, *V*_bias2_, …, *V*_bias1_, *V*_bias2_), which contains 31 pairs of configuration (*V*_bias1_, *V*_bias2_). Each channel is tuning from 0 V to 10 V, with the step of 0.2 V. The *S*_11_ parameters are obtained by connecting the TX horn antenna to a vector network analyzer (Keysight N5230C). After the calibration, the 62 programmable DC output of adaptive feedback control module are connecting to the bias lines of the metasurface. The TX horn antenna is connected to a microwave signal generator (Keysight E8267D), which provides the excitation signal at a fixed frequency. The RX horn antenna is used to receive the scattered signals via a spectrum analyzer (Keysight E4447A). Then the MCU (STM32F103CBT6) is exploited to control DACs and driver circuits to generate dynamic biasing voltages for the metasurface based on to the calibration. The adopted MCU is a low-cost system with the clock speed of 72 MHz, in which a code is preloaded to control the 62-channel DACs generating biasing voltages according to the captured frequency information through the data acquisition card with the sampling rate of 1 kHz. Assuming the varactor diodes in each column respond simultaneously, it is possible to calculate that the whole process from sensing to feedback wave regulation is practically in real-time with approximately 1.6 ms. In addition, in order to better exhibit the absorption, reflection and the comparisons between the two functions, we give the frequency band response by measuring the *S*_11_ parameter through the vector network analyzer (Keysight N5230C). A copper plate with the same size of metasurface sample is measured and the reflection spectrum is used for the comparison and normalization.

#### 2.4.4 Experimental results of self-designed/customized functions

In order to verify the metasurface with high frequency-recognition resolution, eleven frequencies (range of 5.36 GHz and 5.56 GHz, with interval of 0.02 GHz) with predefined EM functions are chosen. The customized frequency dependent EM functions are listed as follows: absorption (5.36 GHz), total reflection (5.38 GHz), −30° deflection (5.40 GHz), random diffusion (5.42 GHz), +30° deflection (5.44 GHz), absorption (5.46 GHz), random diffusion (5.48 GHz), total reflection (5.50 GHz), absorption (5.52 GHz), total reflection (5.54 GHz), and absorption (5.56 GHz). For the case of absorption and reflection, the metasurface can only use the 1 bit amplitude capacity of the meta-atom, without considering the phase-coding. It means the coding sequence contains only one state: amplitude “0” for absorption and “1” for reflection. i.e. the coding state sequence of the 31-column controlled metasurface for the absorption at 5.36 GHz is “00000000/00000000/00000000/0000000”, and the one for the reflection at 5.38 GHz is “11111111/11111111/11111111/1111111”. While for the beam scattering patterns control (deflection or diffusion), the four different phases (phase “00”, “01”, “10” and “11”) with amplitude “1” are required in the 2 bit phase control coding sequence. The four phases are corresponding to four states (‘0’, ‘1’, ‘2’, ‘3’). Therefore, according to the coding principle in Reference [33], the coding state sequence for the deflection to −30° at 5.40 GHz is “11003322/11000332/21100332/2111003” and the one for the random diffusion at 5.42 GHz is “00332001/30002221/21202012/3013111”. Finally, the coding state sequences are converted into DC bias voltages loading at the varactor diodes. Details on the bias voltage configurations and coding state sequences of self-defined EM functions implemented for each frequency are provided in Table S2 (supplementary note 5). In addition, the DC bias voltages loaded at the varactors are calibrated before the experiment.

**Figure 7:**
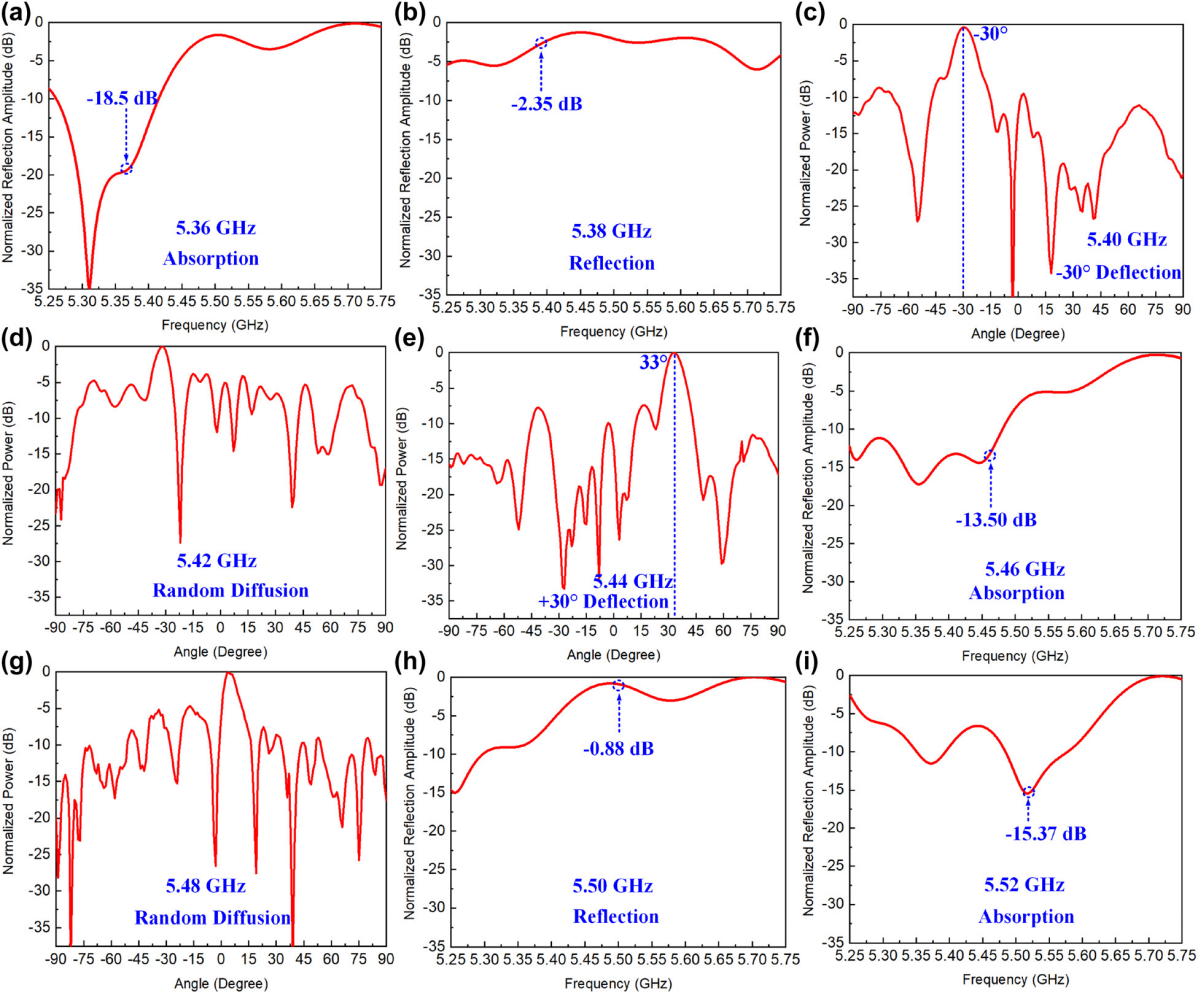
Experimental results. (a)–(i) Measured self-defined EM functions (normalized scattering patterns or reflection spectrum) at different frequencies from 5.36 GHz to 5.52 GHz, respectively.

For assessing the metasurface efficiency of absorption and reflection, the measured *S* parameter (*S*_11_) is normalized by a copper plate with same size. Then the efficiency of the reflection is evaluated by defining reflection (*R* = |*S*_11_|). For assessing the absorption, it can be calculated by using absorption rate (*A* = 1 − *R* ^2^ = 1 − |*S*_11_|^2^). While for the beam deflection at the self-defined frequencies, the efficiency evaluation is carried out by using normalized scattering patterns. Therefore, the absorption rate can be achieved as 98.59%, 95.53%, 97.12%, 99.05% at 5.36, 5.46, 5.52 and 5.56 GHz, respectively (Figures 7(a), (f) and (i) and S6(b)). The maximum absorption appears at 5.56 GHz and all the absorption rates of the four frequencies exceed 95%. For the reflection efficiency, the measured reflection can achieve as high as 90.37% at 5.50 GHz (Figure 7(h)). And the reflections are 76.3% and 78.07% at 5.38 and 5.54 GHz, respectively (Figures 7(b) and S6(a)). Figure 7(c) and (e) show the beam scattering patterns of reflection with different coding sequences illustrated in Table S2. It can be observed that a single directive beam is radiated and the scattering angle is about −30° and 33° deflecting from the normal of metasurface sample in the azimuth plane, which is in very good agreement with the design targets (−30° and 30° deflection). For the case of beam diffusion, the elements of digital phase response are randomly selected to generate the disorder scattering under the frequencies of 5.52 and 5.58 GHz respectively, and the backward scattering has been reduced accordingly (Figure 7(d) and (g)). These demonstrate that the frequency agile functions are realized self-adaptively with sensing the frequencies of the incoming waves applying this intelligent system.

Nevertheless, there are still three aspects to be considered to improve this research: (1) in our work, the meta-atom of the metasurface is designed for *y*-polarization wave and we consider only the incident wave of single linear polarization. Therefore, the proposed metasurface can sense and control the *y*-polarization wave. The design of dual polarization and polarization-insensitive metasurface will be researched in the further study. (2) It is the first trial to design reflective metasurface with independently controlled amplitude and phase. At the beginning, the authors also attempted to design 2 bit phase coding and 2 bit amplitude coding. However, it is challenging and not easy to achieve. Because this is a validating design, and the main purpose is to verify our design principle of frequency-recognition metasurface through the prototype and experimentation. So we presented an active meta-atom to reach 2 bit phase coding and 1 bit amplitude coding capacities to control the amplitude and phase independently. Hence the design of meta-atom with two-bit and more-bit phase and amplitude independent coding needs to be further researched. (3) We use the column controlled metasurface and consider only the incident wave changing along *y* direction. If the metasurface can be controlled in two-dimensional, it can realize regulation of the reflection wave along both *x* and *y* direction. However, it will increase the system complexity due to the feeding network and control system. In the future study, the pixel-level tuned metasurface will be considered for realize more electromagnetic functions, such as holography and orbital angular momentum wave.

## 3 Conclusions

We propose an intelligent metasurface with frequency recognition which can self-adaptively manipulate the spatial EM waves with arbitrary self-defined functions under the agile frequencies. Integrated with a real-time radio frequency sensing and adaptive feedback control electronic system, the customized frequency dependent functions of automatic absorption, reflection, beam deflection and diffusion can be achieved with accurately recognizing the frequencies of incoming waves correspondingly. A programmable reflective metasurface with independent control of phase and amplitude was designed, fabricated and measured. It has digital coding capability of independent 2 bit phase and 1 bit amplitude. The experimental results demonstrate that the intelligent frequency-recognition metasurface system can self-adaptively and accurately manipulate the spatial reflected EM waves in response to different frequencies of incident waves. Our concept can bring latent abilities in single-sensor imaging, advanced radar and communication systems.

## Supplementary Material

Supplementary Material
