# Supplementary material for: Intelligent metasurface with frequency recognition for adaptive manipulation of electromagnetic wave
Source: Nanophotonics. 2022 Mar 7;11(7):1401–11. doi: 10.1515/nanoph-2021-0799 (PMC11501168; doi:10.1515/nanoph-2021-0799)
Supplement: Supplementary file 1 — Supplementary Material [file j_nanoph-2021-0799_suppl.doc]

**Supplementary Information for**

Intelligent Metasurface with Frequency Recognition for Adaptive Manipulation of Electromagnetic Wave

Hai Peng Wang^1,2^, Yun Bo Li^1^*, He Li^1^, Jia Lin Shen^1^, Shu Yue Dong^1^, Shi Yu Wang^1^, Kai Nan Qi^3^, Qian Ma^1^, Shi Jin^2^*, Si Jia Li^1,4^& Tie Jun Cui^1^*

^1^State Key Laboratory of Millimeter Waves, Southeast University, Nanjing 210096, China ^2^National Mobile Communications Research Laboratory, Southeast University, Nanjing 210096, China

^3^Science and Technology on Electromagnetic Scattering Laboratory, Beijing 100854, China

^4^Information and Navigation College, Air Force Engineering University, Xi’an 710077, China

^*^ E-mail: 101012441@seu.edu.cn, (Y.B.Li), tjcui@seu.edu.cn (T.J.Cui), and jinshi@seu.edu.cn (S.Jin)

**Supplementary Note 1: Design of the meta-atom**

According to the depicted geometrical structure of the meta-atom (Fig. 2a and Fig. S1a), two metallic split-square-ring patches and two inner rings are etched on the top layer of the F4BTM220 dielectric substrate (relative permittivity *ε*_r_ = 2.2) with thickness of 0.254 mm. A full metal sheet is placed at the bottom of the F4BTM265 dielectric substrate (*ε*_r_ = 2.65) with thickness of 1.7 mm. The above two kinds of dielectric substrates are bonded by a RO4450F film (*ε*_r_ = 3.52) with thickness of 0.1 mm. Two microwave varactor diodes (D_1_, D_3_) are parallel inserted between gaps of two metallic split-square-ring patches. Another varactor (D_2_) is inserted into the gap between two inner rings with meander line structure. One pole of the varactor is grounded through a metallized via hole, and the other one is connected to a DC bias line etched on the bottom layer of the F4BTM220 dielectric substrate. The metal sheet of the meta-atom was 0.018 mm-thick copper. In addition, the geometrical parameters of the top pattern are optimized with a great number of simulations. The parameters are as follows: *d* = 13 mm, *a* = 12 mm, *b* = 1.5 mm, *l* = 6.6 mm.

In the full wave numerical simulation, the commercial EM software ANSYS HFSS 2018 is used. The boundary is set to be master and slave, and the excitation is set as Floquet port. In this work, the MA46H120 commercial varactor diode manufactured by MA-COM Technology Solutions Inc. is selected, whose SPICE model is illustrated in Supplementary Fig. S1(b). For simplicity, the equivalent series lumped-parameter circuit model shown in Fig. S1(c) is used in the HFSS simulation. The varactor capacitance *C*_T_ can be tuned between 0.17 pF and 1.1 pF with a series parasitic resistance *R*_s_ of 2 Ω [S1].


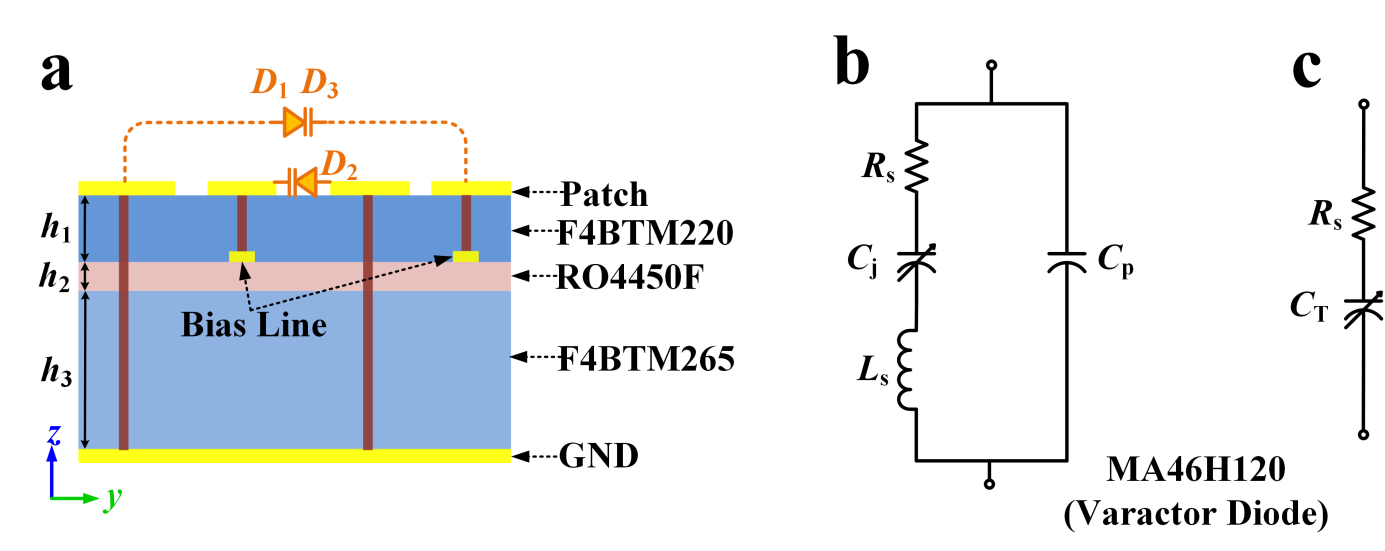


**Figure S1 | The meta-atom and the modeling of the varactor diode. (a)** The topological structure of the proposed meta-atom unit. **(b)** The SPICE model of the MA46H120 varactor diode. **(c)** The equivalent circuit model used to represent the varactor diode in the full-wave simulation.

**Supplementary Note 2: Design and experimental test of radio frequency sensing module**

The frequency sensing module is consists of a low noise amplifier (LNA), three frequency dividers, a precision frequency-to-voltage converter and an analog-to-digital converter (ADC). (Fig. 4). The RF signal is received from the central meta-atom in the metasurface and goes through the frequency sensing module via the SMA connector. A commercial and active LNA module with a gain of 40 dB is employed, and it has a broadband input frequency range from 3.1 to 10.6 GHz. The frequency divider part is composed of divide-by-4, divide-by-256, and divide-by-400 frequency dividers. The schematics of the divide-by-4 and divide-by-400 frequency dividers are shown in Figs. S2a, b, respectively. The active chip HMC433 is a low noise divide-by-4 static divider MMIC with the input frequency range from DC to 8 GHz and a minimum input power level of -12 dBm. The power level of the received RF signal is very low, so the received RF signal is then sent to the LNA, which increase and set the appropriate power level (*LNA_RF*_out_) going into the frequency divider. A commercial module is selected as divide-by-256 prescaler with the input frequency of 1.25 GHz to 1.625 GHz. Then the RF signal (*DIV4_RF*_out_) can be down-converted to the signal (*DIV256_F*_out_) with the frequency range of 4.88 MHz to 6.35 MHz. After going through the divide-by-400 frequency divider, which is composed of digital logic D-type triggers (74HCS74) and counters (74ALS168), the frequency of the signal (*DIV256_F*_out_) is divided into the signal (*DIV_F*_out_) with the low frequency range of 12.2 kHz to 15.875 kHz. Finally, a precision F-to-V converter (LM331) and a non-inverting amplifier (LMV321) are used to generate the final output DC voltage (Fig. S3c), and it has a linear relationship with the frequency of input signal (*DIV_F*_out_).

$V_{out}=DIV\_F_{out}\cdot\frac{2.09\cdot R_{t}\cdot C_{t}\cdot R_{L}}{R_{s}}\cdot(1+\frac{R_{f1}}{R_{f2}})$ (S1)

Then, based on Eq. (S1) and the frequency division, the linear relationship between the input RF frequency (*RF*_in_) from the metasurface and the final output DC voltage (*V*_out_) of frequency sensing module can be given as:

$V_{out}=\frac{RF_{in}}{4\cdot256\cdot400}\cdot\frac{2.09\cdot R_{t}\cdot C_{t}\cdot R_{L}}{R_{s}}\cdot(1+\frac{R_{f1}}{R_{f2}})$ (S2)

Therefore, the frequency information of the monochromatic incident wave can be easily obtained by using the digital sampling with an analog-to-digital converter (ADC) in the data acquisition card (NI USB-6009). Detailed *RC* parameters of the frequency sensing module shown in Fig. S2 are provided in Table S1.


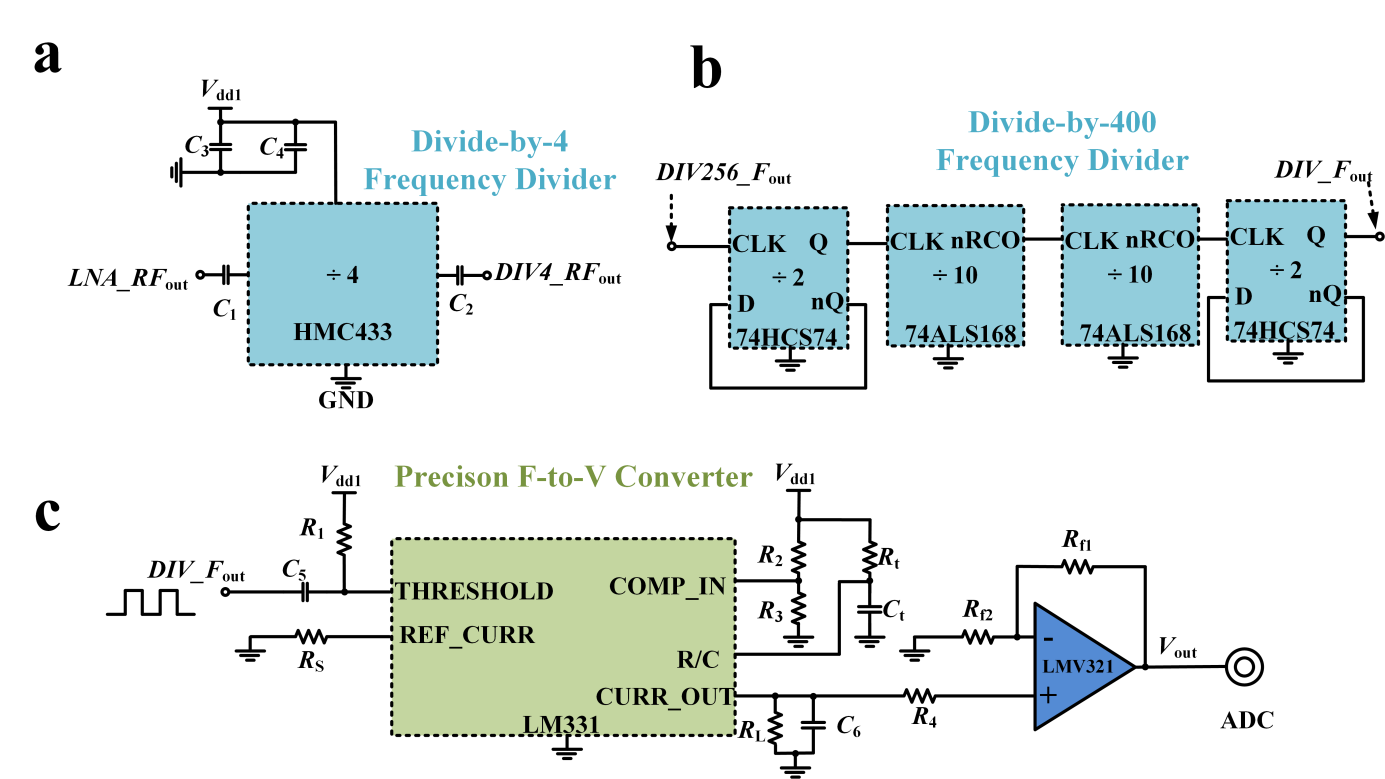


**Figure S2 | Schematic of radio frequency sensing module. (a)** Schematic of the divide-by-4 frequency divider. **(b)** Schematic of the divide-by-400 frequency divider**.** **(c)** Shematic of precision frequency-to-voltage converter and non-inverting amplifier.

**Table S1.** Detailed RC parameters of the frequency sensing module shown in Fig. S3.

| Parameter | *C*_1_ | *C*_2_ | *C*_3_ | *C*_4_ | *C*_5_ | *C*_6_ | *C*_t_ | *R*_1_ |
| --- | --- | --- | --- | --- | --- | --- | --- | --- |
| Value | 100pF | 100pF | 10uF | 1nF | 470pF | 1uF | 4.7nF | 10kΩ |
| Parameter | *R*_2_ | *R*_3_ | *R*_4_ | *R*_f1_ | *R*_f2_ |  |  |  |
| Value | 10kΩ | 15kΩ | 10kΩ | 10kΩ | 10kΩ |  |  |  |

The frequency sensing module is experimental tested under two test conditions. One condition is the circuit-only test condition, which means the frequency sensing module is connecting with signal source (inserting attenuators). Another is metasurface-working condition, and the frequency sensing module is directly connected with metasurface sample, which has the 1m distance from the transmitting horn connecting with signal source. The test results are shown in Fig. S4. We offer *DIV256_F*_out_ signal with the frequency of 4.88 MHz to 6.35 MHz, the output DC voltage (V_out_) of frequency sensing module is obtained with the range of 2.34V and 3.105V under the test of the divide-by-400 frequency divider, F-to-V converter and non-inverting amplifier. Then the curve between the input frequency *RF*_in_ and the output DC voltage V_out_ of frequency sensing module can be computed according to the Eq. (S2) and is presented in Fig. S4a. Under the metasurface-working condition, the measured curve between the incident frequency *RF*_in_ receiving from metasurface and output DC voltage V_out_ using frequency sensing module is illustrated in Fig. S3b. The blue curve and red curve are the maximum and minimum values of the measured output DC voltage signal for the same incident frequency. It can be observed that there is no overlap of the output DC voltage for the neighboring incident frequency at 5.44 GHz, 5.46 GHz, and 5.48 GHz (Fig. S3b). Therefore, the experimental results verified that our designed frequency sensing module can detect the frequency band from 5 to 6 GHz with a quite high resolution of 0.02 GHz. It offers an authentic input for the adaptive and accurate manipulation of spatial electromagnetic spectrum using intelligent frequency sensing metasurface.


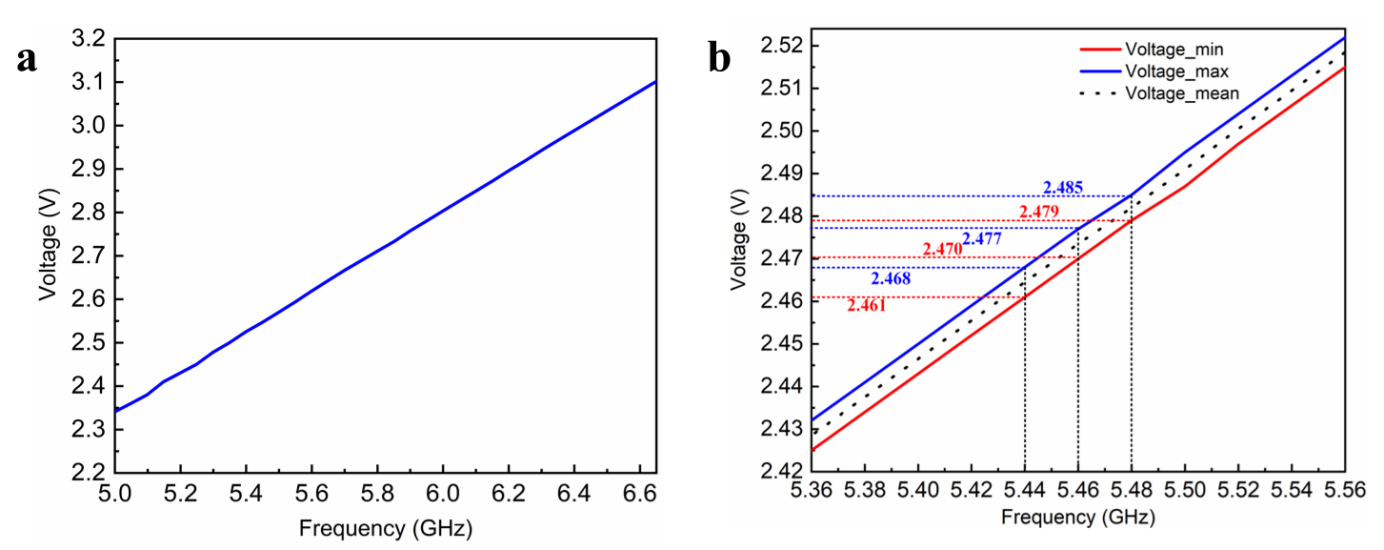


**Figure S3 | Experimental test of the frequency sensing module. (a)** Computed curve between the input frequency (*RF*_in_) and the output DC voltage (V_out_) of frequency sensing module based on the test of divide-by-400 frequency divider, F-to-V converter and non-inverting amplifier. **(b)** Measured curve between the incident radio frequency (*RF*_in_) and the output DC voltage (V_out_) using frequency sensing module under the metasurface-working condition. The blue curve and red curve are the maximum and minimum values of the measured output DC voltage signal for the same incident frequency.

**Supplementary Note 3: Design of the adaptive feedback control module**

The whole programmable metasurface is controlled with a micro-controller unit (MCU), as shown in the Fig. S4a. When the frequency information is obtained by the data acquisition card and the LabView software running in the personal computer (PC), it is transmitted to the MCU through the serial communication port with the baud rate of 115200 bps. So the time of sensing one incoming frequency of wave requires nearly 1.5ms. Then the MCU chip (STM32F103) is used to distribute all commands to the 1394 varcator diodes according to the frequency information. Since each column of metasurface requires two independent bias lines to drive the diodes, the control module needs supply 62 channels of DC signal supply in total. From Fig. S4a, the chip TLV5629 is selected as the digital-to-analog converter (DAC) and has 8 channels of analog output with 8-bit resolution in the output voltage range of 0 and 3.3V. Therefore, 8 digital-to-analog converters (DACs) are employed to generate maximum 64 channels of programmable DC sources. With the use of sharing serial peripheral interface (SPI) and the independent frame synchronization (FS) signals, eight DAC chips are sequentially controlled. Then MCU will send the commands over 62 independent DAC channels, and manipulate the loaded varactor diodes in each column, with the aid of driver circuit connecting with each DAC channel (Fig. S4b). In addition, 62 blue-color LEDs are soldered to indicate the working status of the corresponding channels. Each 8-bit DAC chip TLV5629 requires 16 SPI clock cycles for each voltage output channels, and it has eight output channels. The SPI clock is 18 MHz, and the total time for sending instruction of 62 channels is 55 ($\approx\frac{1}{18}\times16\times62$) μs. Assuming the varactor diodes in each column respond simultaneously, the total time for the whole process from sensing to wave regulation requires approximately 1.6 ms.


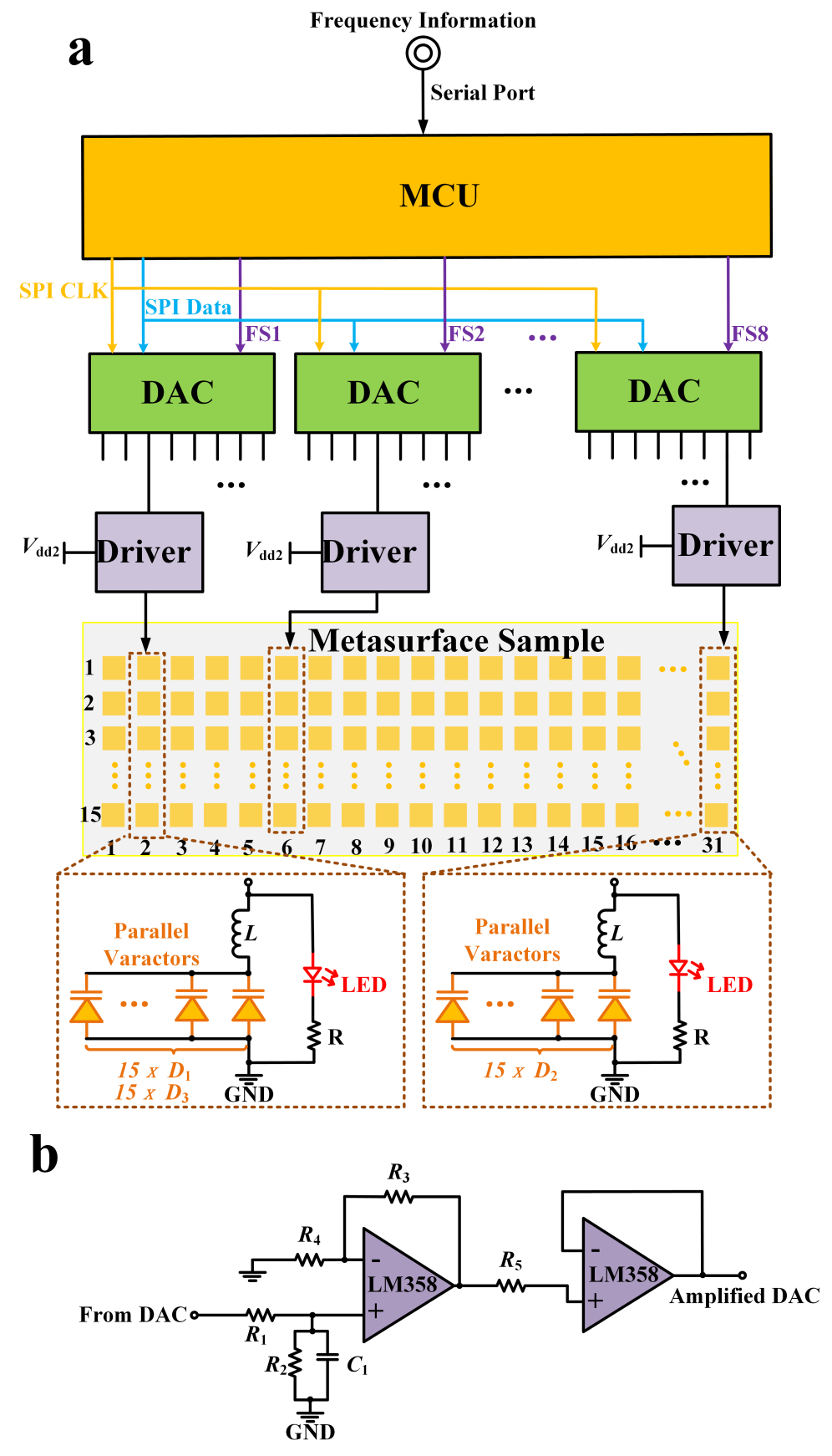


**Figure S4 | Controlling scheme of the feedback control module. (a)** The architecture of the adaptive feedback control module based on MCU. **(b)** The schematic of driver circuit.

**Supplementary Note 4: Reflection spectrum measurement of the meta-atom using standard waveguide**

To verify the reflection characteristics of the designed meta-atom, a sample is fabricated and measured (Fig. S5a). The experimental setup is shown in Fig. S5b, where a standard waveguide WR-159 is used. A waveguide to unit transition component (inset in Fig. S5b) is designed with the thickness of 3mm (~0.055λ). It is an accessory part which is 3D printed and converts the waveguide port size to match the unit size. The device under test (DUT) contains four same meta-unit cells and is surrounded by via holes as substrate integrated waveguide to imitate perfect electric conductor (PEC) boundary. Two DC bias are added through 1nH inductance, which is applied as RF chock. In addition, the bias lines should be isolated with slot through the boundary of the metal sheet to avoid the circuit shorting. The four unit cells are controlled at the same time with consistent bias voltage configurations. Two DC bias voltage sources are provided by a programmable voltage source instrument (NI PXIe-6739R). The cathodes of the varactor diode D_1_ and D_3_ are connected with bias V_bias1_, and the cathode of the varactor diode D_2_ is connected with bias V_bias2_. When the EM wave emits from the waveguide, it passes through the ad-hoc transition first, and returns to the waveguide through the transition after the reflection from the DUT. The S parameters in the vector network analyzer (VNA) are varied by tuning the DC sources. The results are acquired after calibration the waveguide with DUT replaced as copper with the same size. The reflection spectrum can be obtained under the DC bias configuration (V_bias1_, V_bias2_) tuning from 0 V to 10 V, with the step of 0.2 V for each bias voltage. The experiment results are shown in the Fig. S5c-f. It can be observed that the absorption frequency can be shifted between 5.4 GHz and 6.4 GHz by tuning the V_bias2_ from 1V to 10V with loading a constant value of V_bias1_ (Fig. S5c,d). The reflection phase of the meta-atom experiences about 90 degree phase difference with the same amplitude (around 0.52) when the varcator diodes are biased at four different combinations (V_bias1_, V_bias2_) in the frequency range from 6.04 to 6.06 GHz (Fig. S5e,f). It demonstrates that the designed meta-atom can simultaneously realize obvious 2-bit phase shifting and 1-bit amplitude control at the given frequency. Similarly with simulation results, we can also realize the 2-bit phase response at a variable frequency through adjusting the DC bias configurations. However, the waveguide experimental results show a significant frequency shift compared with simulation results. We consider the reason mainly include three aspects. Firstly, it is mainly due to the impinging EM wave to the surface of unit under oblique incidence in the standard waveguide. Secondly, the boundary is set as master and the slave boundary in the HFSS simulation, while the surrounding via holes imitating the PEC is adopted as the boundary in the real measurement. Furthermore, the used transition component also introduces some errors to the experiment.


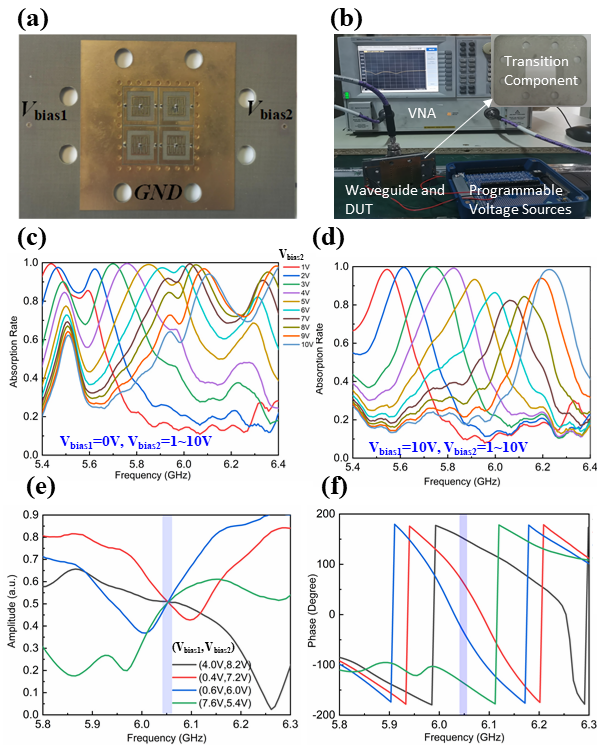


**Figure S5 | Reflection spectrum measurement of the meta-atom using standard waveguide. (a)** The top view of the fabricated meta-atom sample for the waveguide-based test. **(b)** The waveguide-based experimental setup of the designed meta-atom. The inset is a designed waveguide to unit transition component **(c)** **(d)** Measured abosorption rate of the designed meta-atom applied by different bias voltages for normal wave incidence (V_bias1_=0V and 10V, and same colour lines in the two subfigures correspond to the same reverse bias voltages V_bias2_). **(e)** **(f)** Measured results of reflction amplitude and phase responses of the designed meta-atom at 6.05GHz. The amplitude is calibrated using a copper plate as a perfect electric conductor (PEC).

**Supplementary Note 5: The coding sequences for the self-designed/customized functions**

There are 31 columns along the y-polarized direction. Therefore, the y-polarized waves can be controlled by changing coding sequence via these column-controlled bias voltages. For each column, there is an independent bias voltage configuration (V_bias1_, V_bias2_) to connect the varactor diodes. For the EM functions of absorption and reflection, the coding sequence contains only one state: amplitude “0” for absorption and “1” for reflection. While for the beam scattering patterns control (deflection or diffusion), the four different phases (phase “00”, “01”, “10” and “11”) with amplitude “1” are required in the 2-bit phase control coding sequence, and the four independent bias voltage configurations (V_bias1_, V_bias2_) are represented as four states (State ‘0’, ‘1’, ‘2’ and ‘3’). Each bias voltage configuration is calibrated to represent one state for the fixed incident frequency. In order to display conveniently, we use a coding state sequence to represent the implemented coding sequence. The detailed customized EM functions implemented for the frequency of the incoming wave, with the corresponding bias voltage configuration, and coding sequence are illustrated in Table S2.

**Table S2.** Detailed customized EM functions implemented for the frequency of the incoming wave, with the corresponding bias voltage configuration, and coding sequence

| Frequency  (GHz) | Customized Function | Bias Voltage Configuration  (V_bias1_, V_bias2_) | Coding State Sequence |
| --- | --- | --- | --- |
| 5.36 | Absorption | (1.6 V,0.2 V) -> State ‘0’ | 00000000 / 00000000 / 00000000 / 0000000 |
| 5.38 | Reflection | (10 V,0 V) -> State ‘1’ | 11111111 / 11111111 / 11111111 / 1111111 |
| 5.40 | -30° Deflection | (7 V,1 V) -> State ‘0’  (1.8 V,3 V) -> State ‘1’  (4.6 V,0 V) -> State ‘2’  (10 V,10 V) -> State ‘3’ | 11003322 / 11000332/ 21100332 / 2111003 |
| 5.42 | Random Diffusion | (10 V,10 V) -> State ‘0’  (5 V,0 V) -> State ‘1’  (0 V,1.8 V) -> State ‘2’  (6.4 V,0.6 V) -> State ‘3’ | 00332001 / 30002221/ 21202012 / 3013111 |
| 5.44 | +30° Deflection | (7.2 V,2 V) -> State ‘0’  (4.2 V,9.4 V) -> State ‘1’  (10 V,0 V) -> State ‘2’  (5.2 V,0.2 V) -> State ‘3’ | 22330011 / 12233001/ 12233001 / 1222330 |
| 5.46 | Absorption | (2.6 V,1.4 V) -> State ‘0’ | 00000000 / 00000000 / 00000000 / 0000000 |
| 5.48 | Random Diffusion | (2.4 V,0.4 V) -> State ‘0’  (5.4 V,0.2 V) -> State ‘1’ | 00111010 / 10011101/ 00100100 / 1010011 |
| 5.50 | Reflection | (3 V,3 V) -> State ‘1’ | 11111111 / 11111111 / 11111111 / 1111111 |
| 5.52 | Absorption | (3 V,2.4 V) -> State ‘0’ | 00000000 / 00000000 / 00000000 / 0000000 |
| 5.54 | Reflection | (3 V,3 V) -> State ‘1’ | 11111111 / 11111111 / 11111111 / 1111111 |
| 5.56 | Absorption | (3 V,3.6 V) -> State ‘0’ | 00000000 / 00000000 / 00000000 / 0000000 |


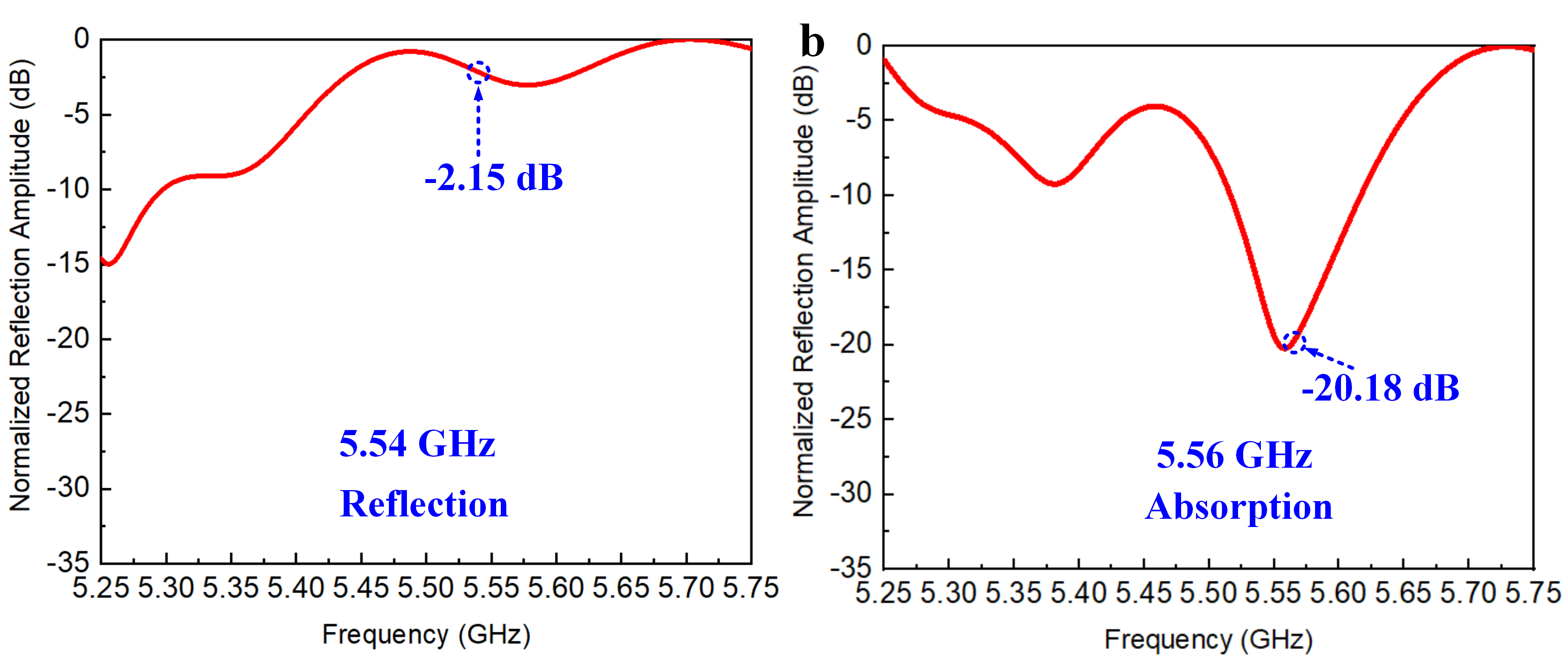


Figure S6 | Additional experimental results of measured self-defined EM functions at different frequencies. (a) 5.54 GHz. (b) 5.56GHz.

**References**

[S1] MA-COM Technology Solutions, MA46H120 Flip Chip varactor Diodes, <http://cdn.macom.com/datasheets/MA46H120.pdf>, (accessed: March, 2017)
